# Berbamine Inhibits Cell Proliferation and Migration and Induces Cell Death of Lung Cancer Cells via Regulating c-Maf, PI3K/Akt, and MDM2-P53 Pathways

**DOI:** 10.1155/2021/5517143

**Published:** 2021-07-08

**Authors:** Lili Liu, Zhiying Xu, Binbin Yu, Li Tao, Ying Cao

**Affiliations:** ^1^Department of Pharmacy, The Affiliated Zhangjiagang Hospital of Soochow University, Zhangjiagang, Jiangsu 215600, China; ^2^Zhangjiagang Fourth People's Hospital, Zhangjiagang, Jiangsu 215600, China

## Abstract

Berbamine (BBM) is a natural product isolated from *Berberis amurensis* Rupr. We investigated the influence of BBM on the cell viability, proliferation, and migration of lung cancer cells and explored the possible mechanisms. The cell viability and proliferation of lung cancer cells were evaluated by MTT assay, EdU assay, and colony formation assay. Migration and invasion abilities of cancer cells were determined through wound scratch assay and Transwell assay. Cell death was evaluated by cell death staining assay and ELISA. The expressions of proteins were evaluated using western blot assay. A xenograft mouse model derived from non-small-cell lung cancer cells was used to detect the effect of BBM on tumor growth and metastasis in vivo. Both colony formation and EdU assays results revealed that BBM (10 *μ*M) significantly inhibited the proliferation of A549 cells (*P* < 0.001). BBM (10 *μ*M) also significantly inhibited the migration and invasion ability of cancer cells in wound scratch and Transwell assays. Trypan blue assay and ELISA revealed that BBM (20 *μ*M) significantly induced cell death of A549 cells. In xenograft mouse models, the tumor volume was significantly smaller in mice treated with BBM (20 mg/kg). The western blotting assay showed that BBM inhibited the PI3K/Akt and MDM2-p53 signaling pathways, and BBM downregulated the expression of c-Maf. Our results show that BBM inhibits proliferation and metastasis and induces cell death of lung cancer cells in vitro and in vivo. These effects may be achieved by BBM reducing the expression of c-Maf and regulating the PI3K/Akt and MDM2-p53 pathways.

## 1. Introduction

Lung cancer is the leading cause of cancer-related death in both men and women. The incidence of lung cancer in China in 2014 was 73.3 per 100,000, ranking the first among malignant tumors, and lung cancer is the leading cause of cancer-related death in both males and females [1]. Non-small-cell lung cancer (NSCLC) accounts for approximately 85% of all lung cancers and is usually diagnosed at an advanced stage with metastasis [2]. Treatments for patients with NSCLC at advanced stage have little therapeutic effect, and relapse often follows cancer therapy. Therefore, new strategies to NSCLC treatment are urgently needed.

Chinese medicine has provided therapeutic options for patients with advanced tumors. Berbamine (BBM) is derived from a traditional Chinese medicine of *Berberis amurensis* Rupr. BBM has been widely used in Asian countries for patients with leukopenia and did not show any obvious side effects [3]. Recent studies have shown that BBM has antioxidation, immunoregulation, and antiarrhythmia activities [4–7]. BBM was also revealed to have potential antitumor function [8–10]. Studies demonstrated effects of BBM on cell activity and migration, with mechanisms involving Bcl-2/Bax, and BBM was also revealed as an inhibitor of Stat3 [11] and NF*κ*B [4, 12, 13]. However, few reports have examined the antitumor effects of BBM on lung cancer. Thus, more studies are needed to study the effects and potential mechanism of BBM in NSCLC.

Abnormality of the PI3K/Akt signaling pathway is related to tumor progression. Aberrant activation of the PI3K/Akt signaling pathway occurs in a wide range of human tumors [2]. Therefore, researchers have investigated therapeutic strategies to target the PI3K/Akt signaling pathway. For example, Shakti et al. found that bergapten inhibited liver carcinogenesis by downregulating the expression of PI3K [14]. Wu et al. found that high expression of PI3K is associated with metastasis and cisplatin resistance in NSCLC [15]. In addition, activation of both the Lyn/PI3K/Akt and MAPK/ERK pathways by green barley extract mitigated the cytotoxicity in human lymphocytes undergoing aggressive oxidative stress [16]. Amplification of PI3K and overaction of Akt or mutations in some of the regulatory components in the PI3K/Akt pathway are the main reasons of metastasis and drug resistance [17]. Thus, better understanding of the PI3K/Akt signaling pathway is helpful to identify potential targets for lung cancer treatment.

MDM2 is a novel downstream factor of PI3K [18]. Elevated MDM2 levels promote ubiquitination and degradation of E-cadherin [12], which in turn promotes cancer cell invasion [19]. The p53 tumor suppressor plays a critical role in regulating cell death, cell cycle arrest, and apoptosis. MDM2 interacts with p53 to promote p53 degradation [20]. c-Maf is a member of the basic leucine zipper transcription factor family [21], and c-Maf is highly expressed in MDA-MB-231 and MCF breast cancer cell lines [19]. Zhang et al. found that knockdown of c-Maf led to the downregulation of MDM2 in human gastric cancer cells [22].

The in vitro study will help to elucidate the BBM's impact on cell viability, proliferation, invasion, and migration in lung cancer cells and indirectly explore BBM's effect on the cell cycle profile. In this study, we examined the potential anticancer effects of BBM on NSCLC. We evaluated the impact of BBM on the cell viability, proliferation, invasion, and migration in lung cancer cells. We also investigated the effect of BBM on tumor growth and metastasis in nude mice and the potential involvement of the PI3K/Akt and MDM2-p53 signaling pathways and c-Maf in the effects of BBM.

## 2. Materials and Methods

### 2.1. Drugs and Reagents

Berbamine dihydrochloride (BBM, purity ≥ 98%) was purchased from Macklin (Shanghai China, cat. no. B860680). PRMI 1640 medium (cat. no. SH3080901) was obtained from Hyclone (America). Dulbecco's modified Eagle's medium (DMEM cat. no. 10-013-CVR) was obtained from Corning (America). Fetal bovine serum (FBS, cat. no. 1624016) was obtained from Gibco (Gibco; Thermo Fisher Scientific, Inc.). Matrigel (cat. no. 356234) was obtained from BD Biosciences (America). Cell Death Detection ELISA Kit (cat. no. 11544675001), LY294002 (cat. no. 934389-88-5), and MTT (cat. no. M5655) were supplied by Sigma-Aldrich (America). The antibodies of PI3K (cat. no. ab70912), MDM2 (cat. no. ab16895), Akt (cat. no. ab8805), p-Akt (cat. no. ab38449), p53 (cat. no. ab38449), and GAPDH (cat. no. ab8245) were purchased from Abcam (England). Bcl-2 (cat. no. sc-578), Bax (cat. no. sc-7480), and c-Maf (cat. no. sc-518062) were purchased from Santa Cruz Biotechnology (America). Caspase-3 (cat. no. 66470-2-1g) and *β*-actin (cat. no. 66009-1-1g) were purchased from Proteintech (America). AMG232 (cat. no. 1352066-68-2) was obtained from MedChemExpress (America). All the other reagents were obtained from Beyotime Biotechnology (Shanghai, China).

### 2.2. Cell Line and Cell Culture

The lung cancer cell line A549 was obtained from the Cell Bank of the Chinese Academy of Sciences (Shanghai, China). PC9 was purchased from BIOWING (Shanghai, China). The A549 cells were cultured with RPMI 1640 medium containing 10% FBS in a humidified atmosphere with 5% CO_2_ at 37°C, and the PC9 cells were cultured with RPMI 1640 medium containing 10% FBS in a humidified atmosphere with 5% CO_2_ at 37°C. When evaluating the anticancer effect of BBM with PI3K inhibitor and MDM2-p53 inhibitor, the cells were pretreated with 1 *μ*M AMG232 for 8 hours [23] and 10 *μ*M LY294002 for 18 hours [24].

### 2.3. MTT Assay

Cell viability was evaluated by MTT assay [25]. Briefly, A549 cells and PC9 cells were cultured in 96-well plates (1 × 10^4^ cells/well) and treated with vehicle (medium with 0.08% DMSO) or BBM (0, 1.25, 2.5, 5, 10, 20, 40, and 80 *μ*M) for 24 h. Then the MTT solution (5 mg/ml) was added to each well, and cells were cultured for 4 h. Then the supernatant was gently removed and 200 *μ*l DMSO was added to each well. The absorbance was detected by a Multiwell Microplate Reader (Bio-Rad Laboratories) at 560 nm.

### 2.4. Colony Formation Assay

Colony formation assay was performed as previously described [26]. A549 cells and PC9 cells were inoculated into 6-well plates and treated with vehicle or different concentrations of BBM (10, 20, and 40 *μ*M) for 24 h. The medium was then gently replaced with complete medium (with 10% FBS). Cells were cultured, and the medium was replaced every 3 days. After 8 days, the cells were stained with crystal violet.

### 2.5. EdU Cell Proliferation Assay

The cell proliferation was evaluated by the EdU Apollo-567 In Vitro Kit (Ribobio, Guangzhou, China, cat. no. C10310-1) [26]. Briefly, A549 cells and PC9 cells (8 × 10^3^ cells/well) were plated into 96-well plates with vehicle or BBM (10, 20, and 40 *μ*M) and cultured for 24 h. Then EdU was added to each well and incubated at 37°C for 2 h. The cell nuclei were stained with Hoechst 33342 at 25°C for 30 min. The ﬂuorescence of cells was observed using an inverted fluorescence microscope (Leica, Germany).

### 2.6. Trypan Blue Staining Assay

Cell death was evaluated by the Trypan Blue staining assay as previously described [27]. A549 cells and PC9 cells (8 × 10^4^ cells/well) were seeded into a 6-well plate and treated with vehicle or different concentrations of BBM (10, 20, and 40 *μ*M). The cells were cultured at 37°C for 48 h. Trypan blue staining was performed according to the manufacturer's instructions. The cells were visualized under a light microscope (Leica DMi 4000).

### 2.7. Cell Death Assay

Cell death was detected using the Cell Death Detection ELISA Kit [28]. This assay is based on the quantitative sandwich immunoassay using antibodies against histones and DNA. The presence of mono- and oligonucleosomes in the cell lysates indirectly represents the apoptosis of cells. A549 cells and PC9 were seeded into the 6-well plates and cultured with or without different concentrations of BBM for 24 h. Then, the RIPA lysis buffer (with 1 mM PMSF) was added to the well, and the supernatant was collected for testing. The mono- and oligonucleosomal fragmented DNA was detected according to the manufacturer's instructions.

### 2.8. Wound Scratch Assay

Cell migration was evaluated by the wound scratch assay [29]. A549 cells and PC9 cells were seeded into a 6-well plate and cultured for 24 h. A 200 *μ*l plastic tip was used to generate a straight line in the cell monolayer. Fresh serum-free medium with vehicle or different concentrations (10, 20, and 40 *μ*M) of BBM were added to each well. The cells were imaged at 0 h and 24 h in the same position of the wound. The migration distance was measured by NIH Image J software. Mitomycin (2 *μ*g/ml) was always added to exclude the proliferation of the cells.

### 2.9. Transwell Assays

Transwell assays [29] were performed to evaluate the metastasis of cancer cells. Briefly, A549 cells and PC9 cells (4 × 10^4^ cells) were seeded into the upper chamber of a Transwell insert (8 *μ*m pore, Corning, America, cat. no. 3412). The cells were allowed to invade or migrate through the chambers at 37°C for 24 h. Cells that invaded or migrated to the surface of the lower chambers were stained and counted. Mitomycin (2 *μ*g/ml) was added in all experiments to exclude the proliferation of cells.

### 2.10. Experimental Animals

Male BALB/c nude mice (6 weeks old, weighing 18–20 g) were obtained from GemPharmatech Co., Ltd. (Jiangsu, China). The mice were housed in polystyrene, well aerated cages with a 12 h light/dark cycle. The animals were maintained on a standard pelleted diet and were provided with free access to food and water *ad libitum*. All studies were performed with the approval of ARRIVE Guidelines (Animal Research: Reporting of In Vivo Experiments) and approved by the Animal Care and Use Committee of Soochow University.

### 2.11. In Vivo Assay

A549 cells were transplanted to the right axillary of the nude mice [30]. When the tumor volume (*W*_2_ ^*∗*^ *L*/2, where *W* = width and *L* = length) reached 150 mm^3^, the mice were randomly divided into three groups: the control group (Ctrl) and the experimental groups (20 mg/kg and 40 mg/kg). Each group had 6 mice. The mice were treated intraperitoneally with isotonic saline or different concentrations of BBM (diluted with isotonic saline) for 10 days, and they were treated daily. The body weight and tumor volume of the mice were recorded every 6 days. At the end of the experiment, the mice were harvested for western blotting assay. The weight and the metastatic nodules of lungs were recorded. The visceral tissues were removed for histopathological examination.

### 2.12. Histopathological Studies

We used the Hematoxylin/Eosin Staining Kit to study the histopathological change of various organs [30]. The tissues were fixed and sectioned at 3-4 *μ*m, and the slices were stained with the Hematoxylin/Eosin Staining Kit (Beyotime, China, cat. no. C0105 M) according to the manufacturer's instructions. Histopathological changes were examined by microscopy (Leica, Germany).

### 2.13. Western Blot

Western blot assay was performed as previously described [13]. Harvested cells or tumors were lysed with RIPA lysis buffer (containing 1 mM PMSF). The protein samples were separated by 10% SDS-PAGE and transferred to PVDF membranes. The membranes were blocked in 5% skim milk for 2 h and then probed with primary antibodies of PI3K (1 : 1000), Akt (1 : 1000), p-Akt (1 : 1000), MDM2 (1 : 500), p53 (1 : 1000), c-Maf (1 : 1000), Bcl-2 (1 : 1000), BAX (1 : 1000), caspase-3 (1 : 1000), *β*-actin (1 : 5000), and GAPDH (1 : 1000) at 4°C, followed by secondary antibodies at room temperature. Bands were detected using the Enhanced ECL Chemiluminescence Detection Kit (Beyotime, China, cat. no. P0018S). Relative band intensity (ratio to GAPDH) was quantified by NIH ImageJ software.

### 2.14. Statistical Analysis

Data are presented as mean ± standard deviation (SD). The statistics were analyzed using SPSS software. Statistical differences were evaluated by Student's *t*-test or one-way ANOVA method; the accepted level of significance was *P* < 0.05.

## 3. Results

### 3.1. Effect of BBM on the Cytotoxicity of Lung Cancer Cells

**Figure 1:**
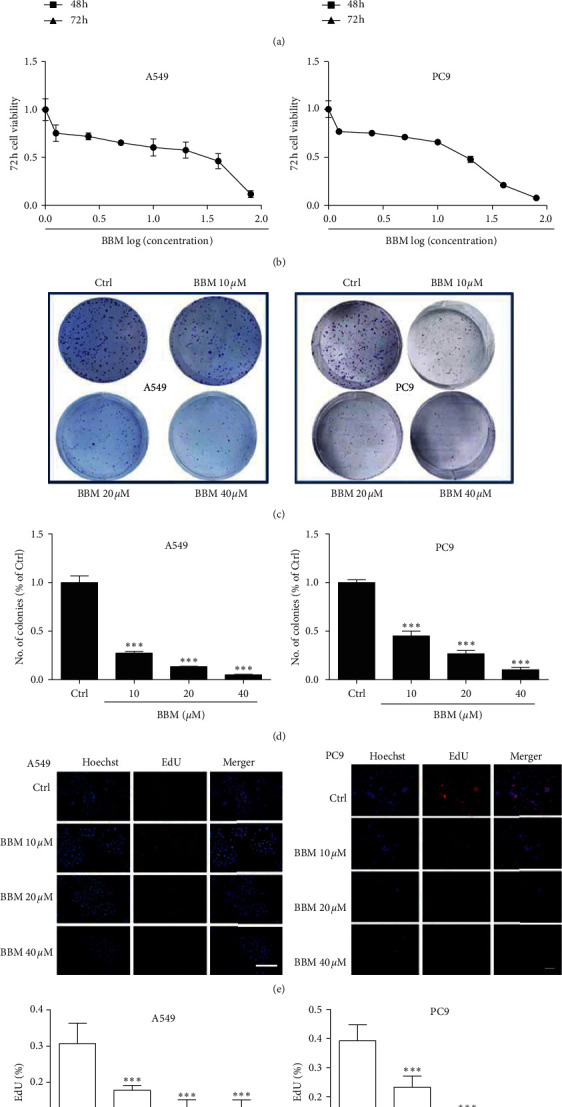
BBM inhibits the cell viability and proliferation of lung cancer cells. Cells were treated with vehicle or various concentrations of BBM, and MTT assays (a, b) (bar = 100 *μ*m in (a)), colony formation assays (c, d) (bar = 100 *μ*m in (c)), and EdU assays (e, f) (bar = 100 *μ*m in (e)) were performed. Data are expressed as mean ± SD (n = 3). ^*∗*^*P* < 0.05 and ^*∗∗∗*^*P* < 0.001 vs. Ctrl group.

The in vitro cytotoxicity of BBM was evaluated by MTT assay, colony formation assay, and EdU assay. The results showed that BBM effectively inhibited the growth of A549 cells and PC9 cells in a time- and dose-dependent manner (Figure 1(a)). The IC_50_ values for BBM against A549 cells and PC9 cells at 72 h were 8.3 ± 1.3 *μ*M and 16.8 ± 0.9 *μ*M, respectively (Figure 1(b)). The results of the colony formation assay showed that colony numbers were significantly decreased among BBM-treated cells compared with the control group (Figure 1(c)). In A549 cells and PC9 cells, BBM at 10, 20, and 40 *μ*M decreased the number of the colonies in a dose-dependent manner (*P* < 0.001; Figure 1(d)). The antiproliferative effect of BBM on lung cancer cells was also observed in EdU assays, and BBM caused a concentration-dependent reduction of proliferation of A549 cells and PC9 cells (Figure 1(e)). BBM at 10, 20, and 40 *μ*M led to a decrease of EdU-positive cells compared with the Ctrl group (*P* < 0.001, Figure 1(f)). Collectively, these results indicated that BBM inhibited the proliferation of lung cancer cells.

### 3.2. Effect of BBM on the Cell Death of Lung Cancer Cells

**Figure 2:**
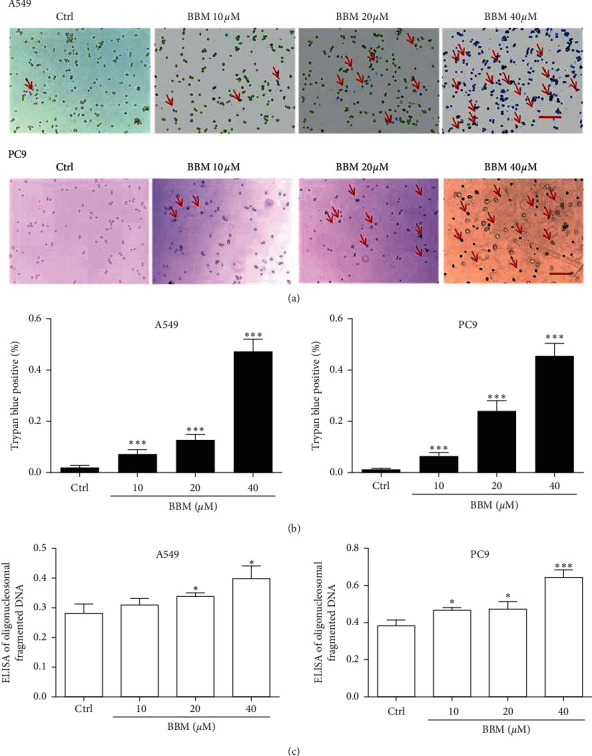
BBM induces cell death of lung cancer cells. Cells were untreated (Ctrl) or treated with BBM. Cell death was detected through Trypan blue assays (a, b) (the arrows indicated that the cells were stained with Trypan blue and bar = 100 *μ*m in (a)) and Cell Death Detection ELISA (c). Data are expressed as mean ± SD (*n* = 3). ^*∗*^*P* < 0.05 and ^*∗∗∗*^*P* < 0.001 vs. Ctrl group.

We examined the effect of BBM on cell death of lung cancer cells using Trypan Blue Dye staining assay and the Cell Death Detection ELISA Kit. The results showed that BBM induced cell death in both A549 cells and PC9 cells (Figure 2(a)). BBM at 10, 20, and 40 *μ*M increased the percentage of Trypan blue-positive in a dose-dependent manner in A549 cells and PC9 cells (*P* < 0.001, Figure 2(b)). These results were further confirmed by an ELISA. BBM at 20 and 40 *μ*M significantly increased the death of A549 cells (*P* < 0.05, Figure 2(c)). BBM at 10, 20, and 40 *μ*M significantly increased the death of PC9 cells (*P* < 0.05, *P* < 0.05, and *P* < 0.001, Figure 2(c)). These results confirmed that BBM induces cell death of A549 cells.

### 3.3. Effect of BBM on Migration and Invasion of Lung Cancer Cells In Vitro

**Figure 3:**
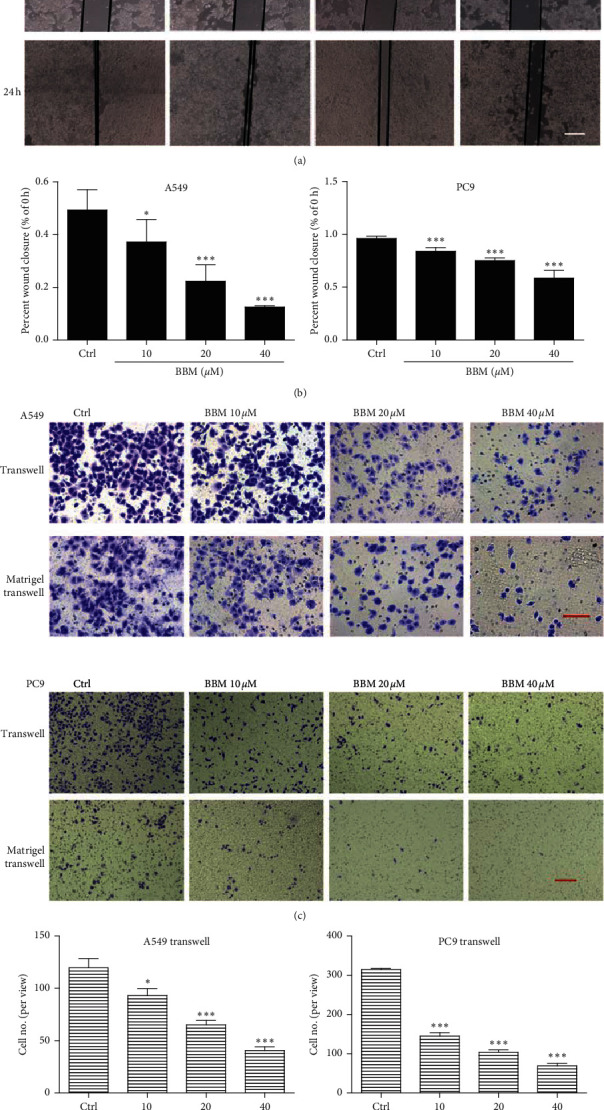
BBM inhibits the migration and invasion of lung cancer cells in vitro. Cells were treated with vehicle or various concentrations of BBM. Cell migration and invasion activities were tested by wound scratch assays (a, b), Transwell assays (c, d), and Transwell assays with Matrigel (c, e). Data are expressed as mean ± SD (*n* = 3). ^*∗*^*P* < 0.05, ^*∗∗*^*P* < 0.01, and ^*∗∗∗*^*P* < 0.001 vs. Ctrl group.

The effects of BBM on the migration and invasion abilities of lung cancer cells were tested by the wound scratch assay and Transwell assay. The results showed that BBM inhibited wound closure in both A549 cells and PC9 cells (Figure 3(a)). BBM at 10, 20, and 40 *μ*M significantly inhibited the migration of A549 cells in a dose-dependent manner (*P* < 0.05, *P* < 0.001, and *P* < 0.001, respectively, Figure 3(b)). BBM at 10, 20, and 40 *μ*M significantly inhibited the migration of PC9 cells in a dose-dependent manner (*P* < 0.001, respectively, Figure 3(b)). Transwell assays further showed that BBM significantly inhibited the migration and invasion of A549 cells and PC9 cells (Figure 3(c)). In migration assays, BBM at 10, 20, and 40 *μ*M decreased the number of A549 cells on the surface of the lower chambers in a dose-dependent manner (*P* < 0.05, *P* < 0.001, and *P* < 0.001, respectively, Figure 3(d)), and BBM at 10, 20, and 40 *μ*M decreased the number of PC9 cells on the surface of the lower chambers in a dose-dependent manner (*P* < 0.001, Figure 3(d)). In invasion assays, BBM at 10, 20, and 40 *μ*M decreased the number of A549 cells on the surface of the lower chambers in a dose-dependent manner (*P* < 0.05, *P* < 0.01, and *P* < 0.001, respectively, Figure 3(e)), and BBM at 10, 20, and 40 *μ*M decreased the number of PC9 cells on the surface of the lower chambers in a dose-dependent manner (*P* < 0.01, *P* < 0.001, and *P* < 0.001, respectively, Figure 3(e)). Together, these results demonstrated that BBM inhibits the migration and invasion of lung cancer cells.

### 3.4. Effect of BBM on the Expressions of c-Maf, PI3K/Akt, and MDM2-p53 Signaling Pathways in Lung Cancer Cells

**Figure 4:**
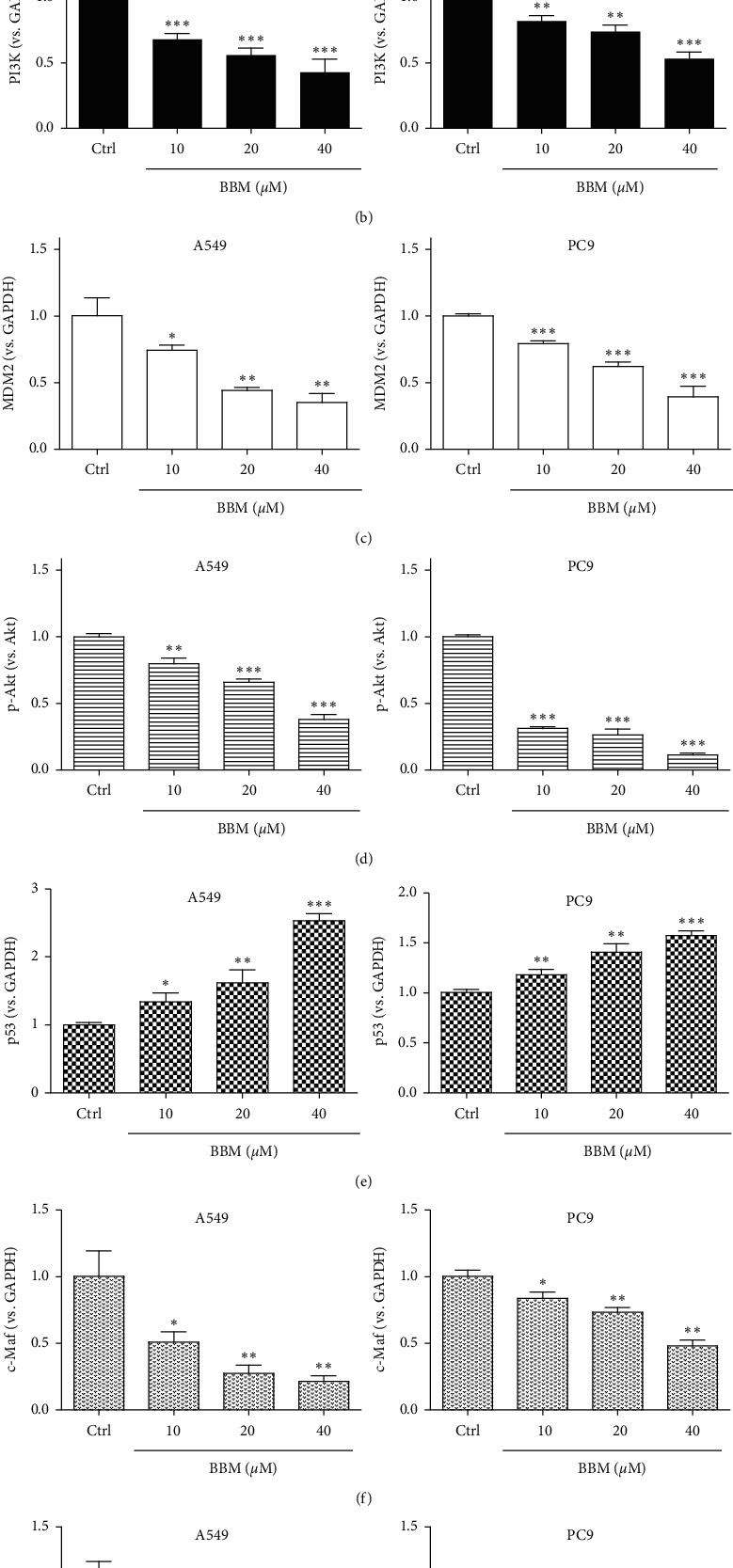
BBM disrupts the PI3K/Akt and MDM2/p53 signal pathways in lung cancer cells. Cells were untreated (Ctrl) or treated with BBM. The expressions of PI3K (a, b), MDM2 (a, c), p-AKT/AKT (a, d), p53 (a, e), c-Maf (a, f), Bcl-2/Bax (a, g), and cleaved-caspase-3/caspase-3 (a, h) were tested by western blot. Data are expressed as mean ± SD (*n* = 3). ^*∗*^*P* < 0.05, ^*∗∗*^*P* < 0.01, and ^*∗∗∗*^*P* < 0.001 vs. Ctrl group.

Since the PI3K/Akt and MDM2-p53 signal pathways are associated with the proliferation, carcinogenesis, and apoptosis of cells, we speculated that BBM may exhibit anticancer effects in lung cancer cells through PI3K/Akt and MDM2-p53 signaling pathways. To examine this hypothesis, we evaluated the expressions of PI3K, MDM2, p-Akt, Akt, p53, caspase-3, c-Maf, Bcl-2, and Bax by western blotting assay. BBM at 10, 20, and 40 *μ*M significantly inhibited the expression of PI3K in A549 cells (*P* < 0.001, Figures 4(a) and 4(b)). BBM at 10, 20, and 40 *μ*M significantly inhibited the expression of PI3K in PC9 cells (*P* < 0.01, *P* < 0.01, and *P* < 0.001, respectively, Figures 4(a) and 4(b)). BBM at 10, 20, and 40 *μ*M significantly inhibited the expression of MDM2 in A549 cells (*P* < 0.05, *P* < 0.01, and *P* < 0.01, respectively, Figures 4(a) and 4(c)). BBM at 10, 20, and 40 *μ*M significantly inhibited the expression of MDM2 in PC9 cells (*P* < 0.001, Figures 4(a) and 4(c)). BBM at 10, 20, and 40 *μ*M significantly inhibited the expression of p-Akt/Akt in A549 cells (*P* < 0.01, *P* < 0.001, and *P* < 0.001, respectively, Figures 4(a) and 4(d)). BBM at 10, 20, and 40 *μ*M significantly inhibited the expression of p-Akt/Akt in PC9 cells (*P* < 0.001, Figures 4(a) and 4(d)). BBM at 10, 20, and 40 *μ*M significantly upregulated the expression of p53 in A549 cells (*P* < 0.05, *P* < 0.01, and *P* < 0.001, respectively, Figures 4(a) and 4(e)). BBM at 10, 20, and 40 *μ*M significantly upregulated the expression of p53 in PC9 cells (*P* < 0.01, *P* < 0.01, and *P* < 0.001, respectively, Figures 4(a) and 4(e)). BBM at 10, 20, and 40 *μ*M significantly inhibited the expression of c-Maf in A549 cells (*P* < 0.05, *P* < 0.01, and *P* < 0.01, respectively, Figures 4(a) and 4(f)). BBM at 10, 20, and 40 *μ*M significantly inhibited the expression of c-Maf in PC9 cells (*P* < 0.05, *P* < 0.01, and *P* < 0.01, respectively, Figures 4(a) and 4(f)). BBM at 20 and 40 *μ*M significantly inhibited the expression of Bcl-2/Bax in A549 cells (*P* < 0.05 and *P* < 0.01, respectively, Figures 4(a) and 4(g)). BBM at 20 and 40 *μ*M significantly inhibited the expression of Bcl-2/Bax in PC9 cells (*P* < 0.05 and *P* < 0.001, respectively, Figures 4(a) and 4(g)). BBM at 40 *μ*M significantly upregulated the expression of cleaved-caspase-3/caspase-3 in A549 cells (*P* < 0.05, Figures 4(a) and 4(h)). BBM at 10, 20, and 40 *μ*M significantly upregulated the expression of cleaved-caspase-3/caspase-3 in PC9 cells (*P* < 0.001, Figures 4(a) and 4(h)).

### 3.5. Effect of PI3K Inhibitor LY294002 and MDM2-p53 Inhibitor AMG232 on the Cell Viability and Metastasis of Lung Cancer Cells

**Figure 5:**
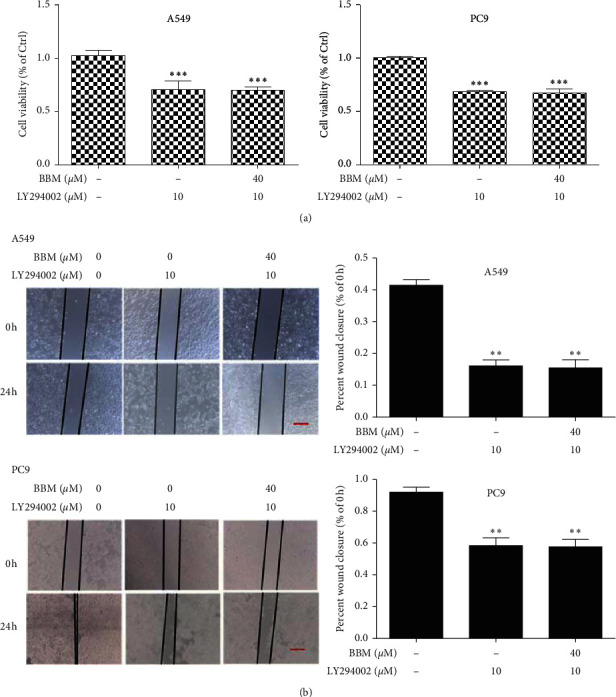
Effects of LY294002 on the cell viability and metastasis of lung cancer cells. Cells were untreated (Ctrl) or treated with LY294002 and BBM. Cell viability was evaluated by MTT assay (a). Metastasis of lung cancer cells was evaluated by wound scratch assay (b) (bar = 100 *μ*m). ^*∗∗*^*P* < 0.01 and ^*∗∗∗*^*P* < 0.001 vs. Ctrl group.

**Figure 6:**
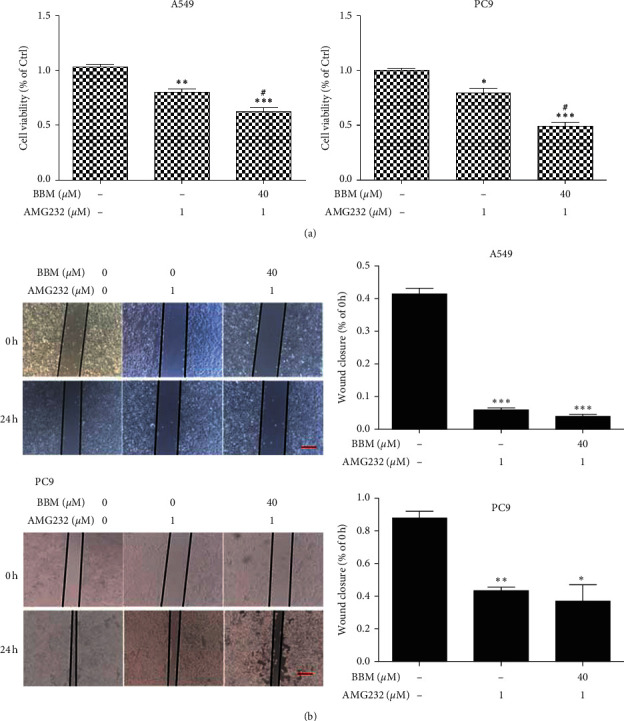
Effects of AMG232 on the cell viability and metastasis of lung cancer cells. Cells were untreated (Ctrl) or treated with AMG232 or BBM. Cell viability was evaluated by MTT assay (a). Metastasis of lung cancer cells was evaluated by wound scratch assay (b) (bar = 100 *μ*m). ^*∗∗*^*P* < 0.01, ^*∗∗∗*^*P* < 0.001 vs. Ctrl group, and #*P* < 0.05 vs. AMG232 group.

We used PI3K inhibitor LY294002 and MDM2-p53 inhibitor AMG232 to observe the anticancer actions of BBM. As the results showed, when the A549 cells and PC9 cells were treated with PI3K inhibitor LY294002, the cell viability was significantly inhibited compared to the Ctrl group (*P* < 0.001, Figure 5(a)), but there was no significant difference between the LY294002 group and the LY294002 + BBM group, and there was no significant difference on migration between the LY294002 group and the LY294002 + BBM group (Figure 5(b)). When the A549 cells and PC9 cells were treated with MDM2-p53 inhibitor AMG232, the cell viability was significantly inhibited compared to the Ctrl group (*P* < 0.01 and *P* < 0.05, Figure 6(a)), and BBM + AMG232 could significantly inhibit the cell viability compared to the AMG232 group (*P* < 0.05, Figure 6(a)), and there was no significant difference on migration between the AMG232 group and the AMG232 + BBM group (Figure 6(b)).

### 3.6. Effect of BBM on the Growth and Metastasis of Tumors In Vivo

**Figure 7:**
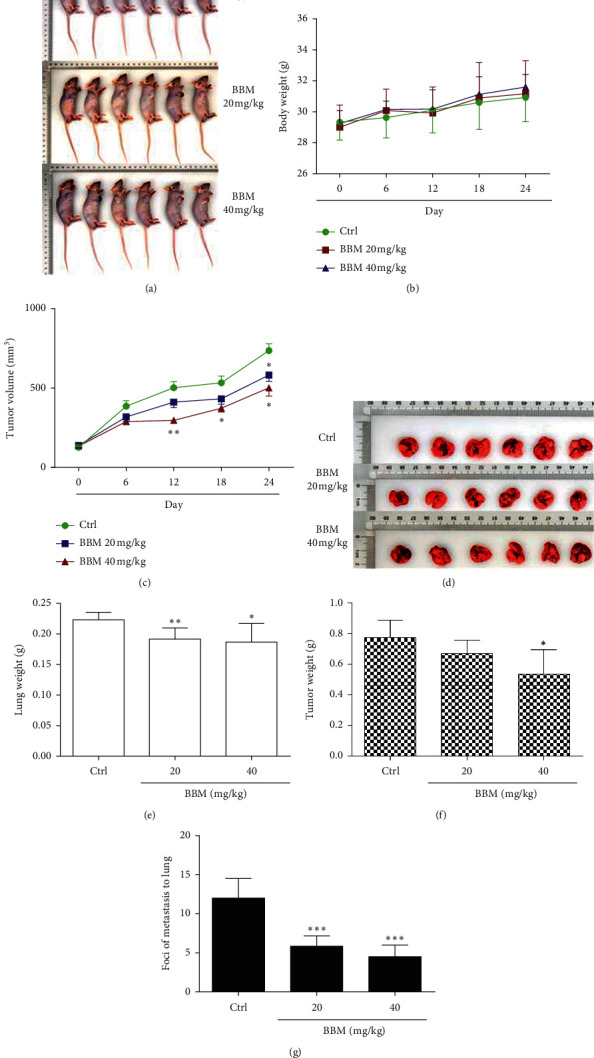
BBM inhibits tumor growth and metastasis in vivo. Nude mice were implanted with A549 cells. When the tumors reached 150 mm^3^, the mice were treated with vehicle (Ctrl) or BBM (a). The body weight of the mice (b) and the tumor volume (c) were recorded. Lung weight (e), tumor weight (f), and the foci of metastasis to lung (g) were measured. Data are expressed as mean ± SD (*n* = 6). ^*∗*^*P* < 0.05, ^*∗∗*^*P* < 0.01, and ^*∗∗∗*^*P* < 0.001 vs. Ctrl group.

**Figure 8:**
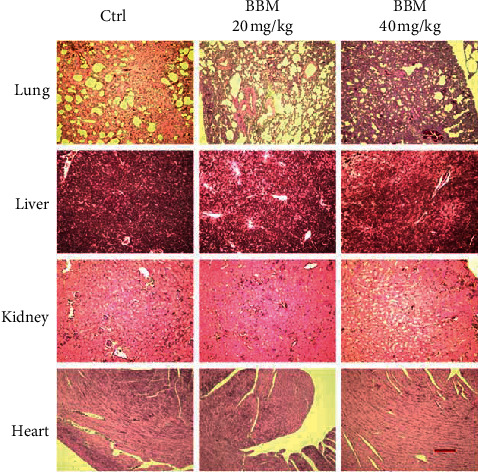
The histopathological changes of lung, liver, kidney, and heart were stained with hematoxylin/eosin and photographed (bar = 100 *μ*m).

To determine the effect of BBM on NSCLC in vivo, we implanted A549 cells into nude mice and treated the mice with BBM or vehicle. The body weight of the mice in the experimental groups showed no significant difference compared with the Ctrl group (Figures 7(a) and 7(b)). The tumor volume of the mice was significantly smaller in mice treated with BBM compared with Ctrl mice (*P* < 0.05, Figures 7(a) and 7(c)). BBM at 20 mg/kg and 40 mg/kg significantly decreased the lung weight (*P* < 0.01 and *P* < 0.05, respectively, Figures 7(d) and 7(e)). The tumor weight in the experimental group (40 mg/kg) was significantly decreased compared with the Ctrl group (*P* < 0.05, Figure 7(f)). In addition, the number of nodules per lung in the BBM group (20 mg/kg and 40 mg/kg) was significantly decreased compared with the Ctrl group (*P* < 0.001, Figure 7(g)). Histopathological evaluations revealed no changes in lungs, livers, hearts, and kidneys between experimental and Ctrl groups (Figure 8).

### 3.7. Effect of BBM on the Expressions of c-Maf, PI3K/Akt, and MDM2-p53 Signaling Pathways In Vivo

**Figure 9:**
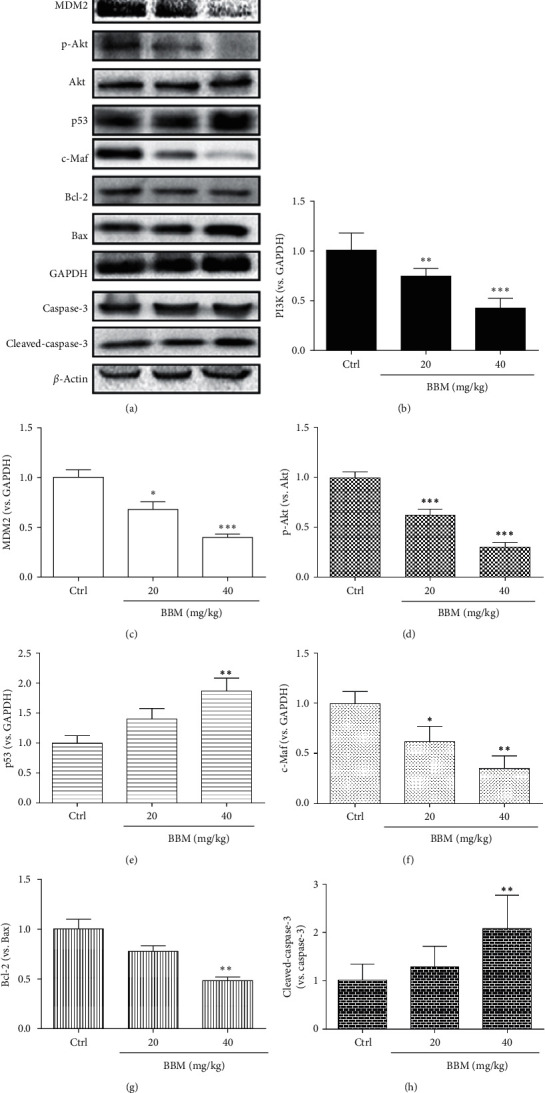
BBM disrupts the PI3K/Akt and MDM2/p53 signal pathways in tumors. ALB/c mice were untreated (Ctrl) or treated with BBM; the expressions of PI3K (a, b), MDM2 (a, c), p-Akt/Akt (a, d), p53 (a, e), c-Maf (a, f), Bcl-2/Bax (a, g), and cleaved-caspase-3/caspase-3 (a, h) were examined by western blot (*n* = 6). ^*∗*^*P* < 0.05, ^*∗∗*^*P* < 0.01, and ^*∗∗∗*^*P* < 0.001 vs. Ctrl group.

MDM2 is an oncoprotein associated with various malignancies; its overexpression is vital for aggressive metastasis [20]. To determine if BBM plays an anticancer role in vivo through the PI3K/Akt and MDM2-p53 signaling pathways, we examined the expressions of PI3K, p-Akt, Akt, MDM2, p53, caspase-3, c-Maf, Bcl-2, and Bax in tumors using western blot assay. BBM at 20 mg/kg and 40 mg/kg significantly reduced the expression of PI3K (*P* < 0.01 and *P* < 0.001, respectively, Figures 9(a) and 9(b)). BBM at 20 mg/kg and 40 mg/kg significantly reduced the expression of MDM2 (*P* < 0.05 and *P* < 0.001, respectively, Figures 9(a) and 9(c)), BBM at 20 mg/kg and 40 mg/kg significantly reduced the expression of p-Akt/Akt (*P* < 0.001, Figures 9(a) and 9(d)). BBM at 40 mg/kg significantly upregulated the expression of p53 (*P* < 0.01, Figures 9(a) and 9(e)). BBM at 20 mg/kg and 40 mg/kg significantly reduced the expression of c-Maf (*P* < 0.05 and *P* < 0.01, respectively, Figures 9(a) and 9(d)). BBM at 40 mg/kg significantly upregulated the expression of Bcl-2/Bax (*P* < 0.01, Figures 9(a) and 9(h)).

## 4. Discussion

Lung cancer is the leading cause of cancer-associated death among both men and women worldwide. In recent years, with the gradual elucidation of the molecular mechanism of NSCLC, more molecular-targeted drugs have been applied in clinical practice and achieved satisfactory results [31, 32]. Therefore, designing new drugs based on key target genes or proteins that play critical roles in NSCLC is important for the treatment of NSCLC in the future.

Previous studies have demonstrated the antitumor effects of BBM in a variety of tumors, including breast cancer [33], myeloma [7, 12], hepatoma [9, 34], prostatic neoplasms [9], pancreatic carcinoma [35], and lung cancer [36]. All these findings indicate the potential of BBM for cancer treatment. Moreover, we demonstrated that BBM inhibited cell proliferation and metastasis of A549 cells and PC9 cells and inhibited migratory and invasive potential of these cells. Xenograft studies were also used to evaluate the effect of BBM on lung cancer. The results indicated that BBM significantly inhibited tumor growth and metastasis without manifesting changes in potential toxic signs such as diarrhea, movement disorders, or swelling, and the histopathological evaluations revealed no changes in lungs, livers, hearts, and kidneys between experimental and Ctrl groups.

The PI3K/Akt pathway is associated with cell survival, invasion, and migration and plays key roles in various cancers [16, 37, 38]. In NSCLC, the PI3K/Akt/mTOR pathway has been heavily implicated in both tumorigenesis and the progression of disease [39]. The phosphorylation of Akt causes further phosphorylation of downstream effectors, such as mTOR and MDM2, which are closely associated with the apoptosis process [40]. Most of the studies showed that natural products exhibited anticancer actions via PI3K/Akt pathway [41]. Furthermore, numerous preclinical studies have shown that some herbs and natural phytochemicals can inhibit AKT activity directly [42, 43]. The effects of BBM on lung cancer cells were deciphered on the alteration in the level of expression of PI3K/Akt-related markers. We also noted that BBM inhibited the expressions of PI3K and Akt. When we used the inhibitor of PI3K, there was no difference in the proliferation between the PI3K inhibitor group and the BBM + PI3K inhibitor group. Thus, PI3K may be the target of BBM on the lung cancer cells, and BBM may inhibit the cell proliferation and metastasis through PI3K/Akt pathway.

MDM2 is an oncoprotein that exhibits dynamic negative regulation of the tumor suppressor p53 [19]. MDM2 is often highly expressed in a variety of human cancers, and its overexpression promotes cancer cell's proliferation [25, 44]. The MDM2-p53 pathway is important in regulating cell events, such as cancer cell death, cell cycle arrest, apoptosis, senescence, and DNA repair [25]. Moreover, MDM2 suppresses the migration of cancer cells and induces apoptosis of cancer cells [29, 44, 45]. Our results showed that BBM inhibited the migration and invasion of A549 cells and PC9 cells in vitro, and the number of nodules on lungs in a xenograft mouse model treated with BBM was significantly reduced in vivo; in addition, the expression of MDM2 was downregulated both in vitro and in vivo. Besides, when we pretreated the cells with MDM2-p53 inhibitor, we found that the migration of the lung cancer cells showed no difference between the MDM2-p53 inhibitor group and the BBM + MDM2-p53 inhibitor group. These results indicate that BBM inhibits cancer cell metastasis and this effect may be achieved by modulating the expression of MDM2.

Recent studies revealed that c-Maf is overexpressed in a variety of cancers including NSCLC [46]. Studies showed that c-Maf is a downstream molecule of PI3K [47] and is indirectly associated with cell migration [48]. Our results showed that the expressions of PI3K and c-Maf were reduced by BBM. Thus, the mechanism of BBM inhibits lung cancer cells maybe through regulating the expression of c-Maf. In addition, Zhang et al. found that CMIP (c-Maf-inducing protein) knockdown downregulated the expression of MDM2 [22]. Divya et al. found that c-Maf was a downstream protein of PI3K/Akt pathway [49]. Our results revealed that migration and invasion were inhibited by BBM both in vitro and in vivo, and cell death was induced by BBM in vitro. The expressions of c-Maf were downregulated by BBM, and the expressions of p53 and cleaved-caspase-3/caspase-3 were upregulated. These results demonstrated that BBM inhibits the migration and induces the cell death of A549 cells. Thus, the expression of MDM2 may be regulated by c-Maf, and p53 may be regulated by MDM2.

## 5. Conclusion

**Figure 10:**
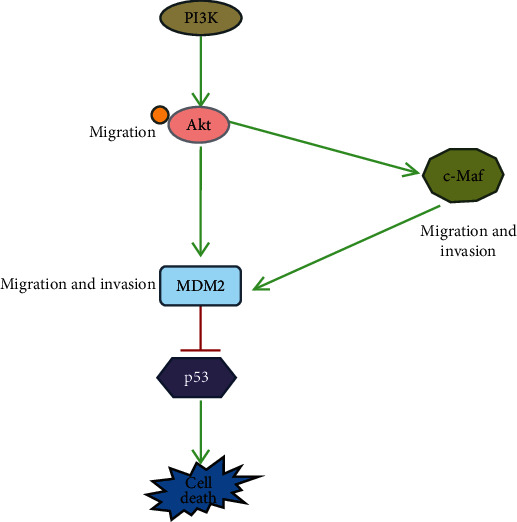
A schematic of the proposed signaling pathway of the current study. BBM downregulates PI3K and Akt and then downregulates the expressions of c-Maf and MDM2, and c-Maf downregulates the expression of MDM2 and eventually upregulates the expression of p53, leading to cell death.

Our study demonstrated that BBM suppresses the proliferation and migration of human lung cancer A549 cells. This effect may be through BBM regulating the expression of c-Maf by blocking the PI3K/Akt signaling pathway. The MDM2-p53 signaling pathway may be regulated by c-Maf (Figure 10). With low toxicity characteristics, exploiting BBM alone or in combination with established chemotherapy regimens may provide a new treatment strategy for lung cancer. However, a noncancerous cell line for the in vitro studies should be included in the future, in particular, investigating whether BBM possesses a favorable selective cytotoxicity index (SCI).

## Data Availability

The data used to support the findings of this study are included within the article.
